# Exploring silique number in *Brassica napus* L.: Genetic and molecular advances for improving yield

**DOI:** 10.1111/pbi.14309

**Published:** 2024-02-22

**Authors:** Hui Wang, Xiaodong Li, Boyu Meng, Yonghai Fan, Shahid Ullah Khan, Mingchao Qian, Minghao Zhang, Haikun Yang, Kun Lu

**Affiliations:** ^1^ Integrative Science Center of Germplasm Creation in Western China (CHONGQING) Science City and Southwest University, College of Agronomy and Biotechnology Southwest University Beibei Chongqing P.R. China; ^2^ Engineering Research Center of South Upland Agriculture, Ministry of Education Chongqing P.R. China; ^3^ Academy of Agricultural Sciences Southwest University Beibei Chongqing P.R. China

**Keywords:** Rapeseed, Silique number, Genetic loci, Molecular mechanisms, Gene regulatory network

## Abstract

Silique number is a crucial yield‐related trait for the genetic enhancement of rapeseed (*Brassica napus* L.). The intricate molecular process governing the regulation of silique number involves various factors. Despite advancements in understanding the mechanisms regulating silique number in *Arabidopsis* (*Arabidopsis thaliana*) and rice (*Oryza sativa*), the molecular processes involved in controlling silique number in rapeseed remain largely unexplored. In this review, we identify candidate genes and review the roles of genes and environmental factors in regulating rapeseed silique number. We use genetic regulatory networks for silique number in *Arabidopsis* and grain number in rice to uncover possible regulatory pathways and molecular mechanisms involved in regulating genes associated with rapeseed silique number. A better understanding of the genetic network regulating silique number in rapeseed will provide a theoretical basis for the genetic improvement of this trait and genetic resources for the molecular breeding of high‐yielding rapeseed.

## Introduction

Rapeseed (*Brassica napus* L., AACC, 2*n* = 38) is a crucial source of edible oil and biofuel and the third largest oil crop globally after oil palm (*Elaeis guineensis*) and soybean (*Glycine max*). Breeders have been improving silique number because it is the trait most highly correlated with yield among the three yield components of rapeseed: silique number, seed number per silique and thousand seed weight (Özer and Oral, 1999). However, silique number is a complex quantitative trait that is susceptible to environmental influences. Hundreds of quantitative trait loci/quantitative trait nucleotides (QTLs/QTNs) for silique number have been identified, but their genetic and molecular bases remain ambiguous (Raboanatahiry *et al*., 2018; Shi *et al*., 2015). Silique number (grain number) in crops is mainly controlled by shoot architecture; the shoot apical meristem (SAM) gives rise to leaf primordia, and the axillary meristem (AM) gives rise to vegetative branches or inflorescences (Wang *et al*., 2018). AMs are established in leaf axils during the vegetative stage and form axillary buds, which subsequently remain dormant or continue to grow and form branches (Bennett and Leyser, 2006). After the transition from the vegetative stage to the reproductive stage, the SAM develops into the inflorescence meristem (IM). The IM then continuously differentiates into the AM and the floral meristem (FM), forming inflorescence branches and floral buds, respectively. These biological processes are influenced by genetics and by environmental conditions such as light, temperature and nutrient status. Therefore, understanding the molecular genetic mechanisms regulating the formation of branches and floral buds is important for the genetic improvement of silique number in rapeseed.

The molecular mechanism controlling the formation of branches and floral buds in plants has been studied extensively (Wang *et al*., 2018; Zhu and Wagner, 2020). Plant hormone signalling and genetic regulation are largely conserved between monocots and dicots (Teichmann and Muhr, 2015; Wang and Jiao, 2018). In general, auxin and strigolactone (SL) inhibit lateral bud growth, whereas cytokinin directly promotes lateral bud growth (Ongaro and Leyser, 2008). Strigolactone synthesis is conserved in plants. Knockout of *MAX1*, a strigolactone synthesis gene, results in increased branching or tillering in *Arabidopsis*, rice and wheat (Cardoso *et al*., 2014; Sigalas *et al*., 2023; Yoneyama *et al*., 2018). The natural auxin indole‐3‐acetic acid (IAA) promotes the expression of *MAX3* and *MAX4* genes in the strigolactone synthesis pathway, inhibiting branches or tillers growth (Domagalska and Leyser, 2011; Hayward *et al*., 2009). Cytokinins can directly promote branches or tillers growth, independent of auxin accumulation (Müller *et al*., 2015). Reduced expression of *OsCKX2* causes cytokinin accumulation in inflorescence meristems and increases the spikelet numbers and rice yield (Ashikari *et al*., 2005). Moreover, numerous genes play crucial roles in the regulation of branching and floral bud formation. For example, *BRANCHED1* (*BRC1*) encodes a TCP transcription factor that promotes bud dormancy but inhibits branch formation by integrating phytohormone and environment signals in most plants (Wang *et al*., 2019). *Lateral repressor* (*Ls*) genes encode members of the VHIID subfamily of plant‐specific GRAS (GA INSENSITIVE, REPRESSOR OF GA1‐3 SCARECROW) transcription factors that are required for AM initiation in many plant species, such as tomato (*Solanum lycopersicum*), *Arabidopsis* (*Arabidopsis thaliana*) and rice (Greb *et al*., 2003; Li *et al*., 2003). Identifying these genetic determinants is helpful for understanding the molecular mechanism regulating silique number and for genetically improving rapeseed.

Here, we summarize the QTLs/QTNs for silique number in rapeseed. We then identify candidate genes for silique number within overlapping QTL/QTN regions. Next, we explore pathways associated with genetic, plant hormonal and environmental stimuli that regulate silique number. By examining gene regulatory networks (GRNs) controlling the formation of branches and floral buds in *Arabidopsis* and rice, we predict the molecular genetic mechanism underlying silique number in rapeseed. This review summarizes our understanding of the genetic regulation of silique number rapeseed, providing a theoretical basis and potential genetic resources for yield improvement in this important oilseed crop.

## Genetic regulation of silique number

### QTLs/QTNs associated with silique number

**Figure 1:**
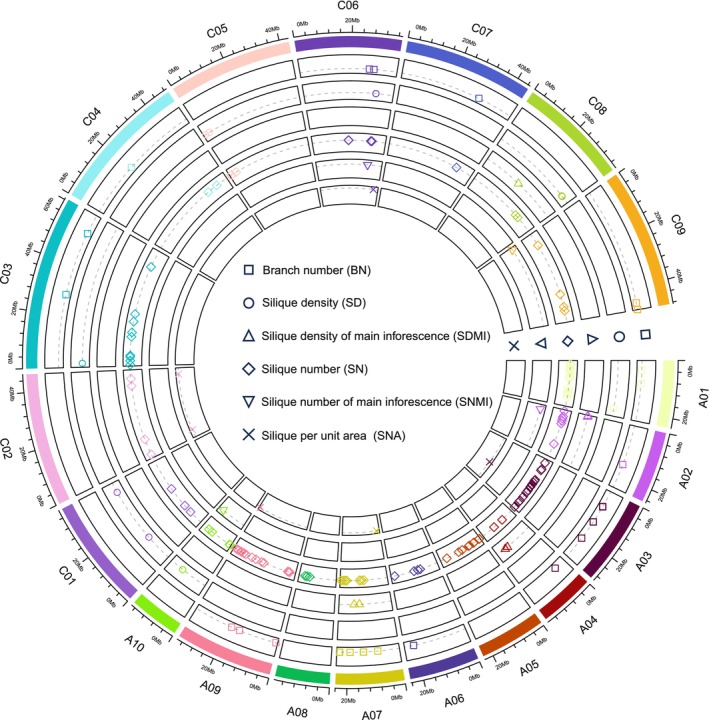
Alignment map displaying regions of QTLs/QTNs for traits related to silique number in rapeseed. From outside to inside, different coloured blocks in the outermost circle represent different genetic linkage groups, the six inner circles represent six traits related to silique number (BN, SD, SDMI, SN, SNMI and SNUA, respectively), and different geometric shapes within the six inner circles with the same colour as their respective blocks represent QTLs/QTNs associated with specific linkage groups identified in previous studies.

**Figure 2:**
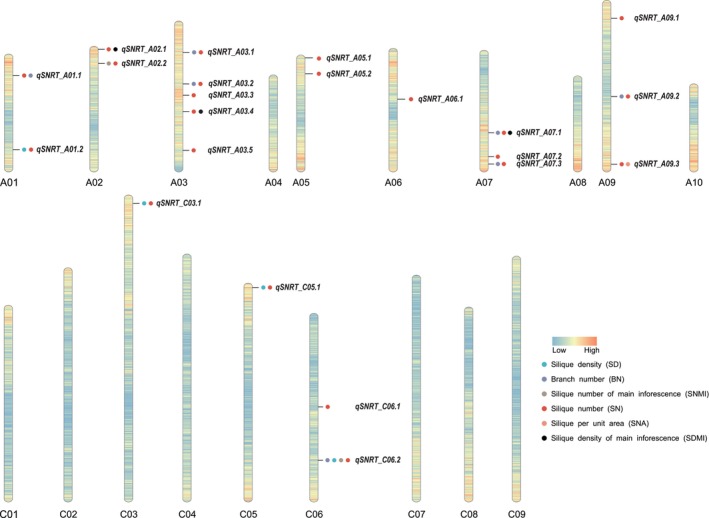
Regions containing overlapping QTLs/QTNs associated with silique number. Overlapping QTLs/QTNs are named *q* + *SNRT* (silique‐number‐related trait) + chromosome + number, and different coloured dots represent different traits related to silique number. Blue to red shading on the chromosomes indicates gene density from low to high.

Silique number is a complex quantitative trait exhibiting a wide range of continuous variation, which is particularly influenced by environmental factors (Xu *et al*., 2014). More than 100 QTLs/QTNs associated with silique number have been identified from at least 10 linkage mapping populations (Cai *et al*., 2014; Ding *et al*., 2012; Qi *et al*., 2014; Radoev *et al*., 2008; Shi *et al*., 2009, 2013; Wang and Guan, 2010; Yi *et al*., 2006). Shi *et al*. (2015) and Raboanatahiry *et al*. (2018) compared multiple QTLs/QTNs for silique number using *B. napus* ‘Darmor‐bzh’ as a reference to explore candidate genes regulating yield and its component traits (Chalhoub *et al*., 2014). Since 2017, 46 additional QTLs/QTNs for silique number have been identified and mapped (Deng *et al*., 2019; Li *et al*., 2020b; Lu *et al*., 2017). Tang *et al*. (2020) mined four major chromosomal regions containing genes with differential transcript abundance in *dpt247* (a recessive mutant with a high density of pods) compared to wild‐type *B. napus*. We refer to these four major chromosomal regions as the major QTLs for silique number. Due to the direct influence of branch number on silique number, QTLs/QTNs for branch number should also be considered. Twenty‐seven QTLs/QTNs for branch number have been identified in seven studies since 2015 (He *et al*., 2017; Li *et al*., 2016, 2020a, 2022a; Liu *et al*., 2021; Luo *et al*., 2015; Zheng *et al*., 2017). In total, 174 QTLs/QTNs for traits related to silique number have been described. We aligned these QTLs with the physical map of the *B. napus* ‘Darmor‐bzh’ reference genome, revealing that these QTLs/QTNs are found in 19 linkage groups (Figure 1; Table S1). Some of the 174 QTLs/QTNs are located in separate regions, while some are overlapped. Several major regions containing overlapping QTLs/QTNs were detected in three or more studies. Twenty‐two regions contain 82 overlapping QTLs/QTNs for silique‐related traits, which are located on chromosomes A01, A02, A03, A05, A06, A07, A09, C03, C05 and C06 (Figure 2; Table S2). These regions range from 1.0 to 7.1 Mb and are associated with one or more traits related to silique number, such as *qSNRT_A03.3* for silique number only and *qSNRT_A03.2* for silique number and branch number. These regions might contain the key genes regulating silique number and its related traits, offering the potential for developing functional markers for marker‐assisted breeding.

### Identification of candidate genes

There are 213 genes in *Arabidopsis* and 248 genes in rice related to silique number or grain number, respectively. We used these 461 genes to identify 2091 homologous genes related to silique number in rapeseed (Shi *et al*., 2009; National Rice Data Center: https://ricedata.cn/; Table S3) using the Browse Data tool at *Brassica napus* multi‐omics information resource (https://yanglab.hzau.edu.cn/BnIR) (Yang *et al*., 2023) and National Rice Data Center. Of 1831 homologous genes with known chromosome locations to uncover 240 candidate genes within twenty‐two regions for traits related to silique number while the remaining 260 orthologues genes could not be used to identify candidate genes since their chromosomal locations were unknown (Figure 2; Tables S2 and S4). These comprised 53 candidate genes for silique number only and 187 candidate genes for multiple traits: 46 for silique number (SN) and branch number (BN); 47 for SN and silique density (SD); 15 for SN and silique number in the main inflorescence (SNMI); 35 for SN and silique number per unit area (SNUA); 11 for SN and silique density of the main inflorescence (SDMI); 24 for SN, BN, and SDMI; and 9 for SN, BN, SNMI and SD. Of the 240 candidates, *BnaC06.TB1* (*TEOSINTE BRANCHED 1*, BnaC06g29550D,), *BnaAP1* (*APETALA1*, BnaA07g24360D, BnaA07g27710D, BnaC06g29980D), *BnaC03.TFL1* (*TERMINAL FLOWER 1*, BnaC03g01440D), *BnaBRC1* (BnaA01g26700D, BnaA03g34820D), *BnaA02.RAX1* (*REGULATOR OF AXILLARY MERISTEMS 1*, BnaA02g06100D) and *BnaREV* (*REVOLUTA*, BnaA02g06170D, BnaA06g18550D) are orthologs of key genes regulating the formation of branches and floral buds, that is *OsTB1*, *AP1* (*OsMADS18*), *TFL1*, *BRC1*, *RAX1* and *REV*, respectively. Some of these candidate genes have been shown to regulate silique number in rapeseed, such as *BnaAP1* (Shah *et al*., 2018) and *BnaTFL1* (Sriboon *et al*., 2020). This supports the likelihood that these genes control traits related to silique number, making them good candidates for further genetic research in rapeseed.

### Genetic control of silique number related traits

**Figure 3:**
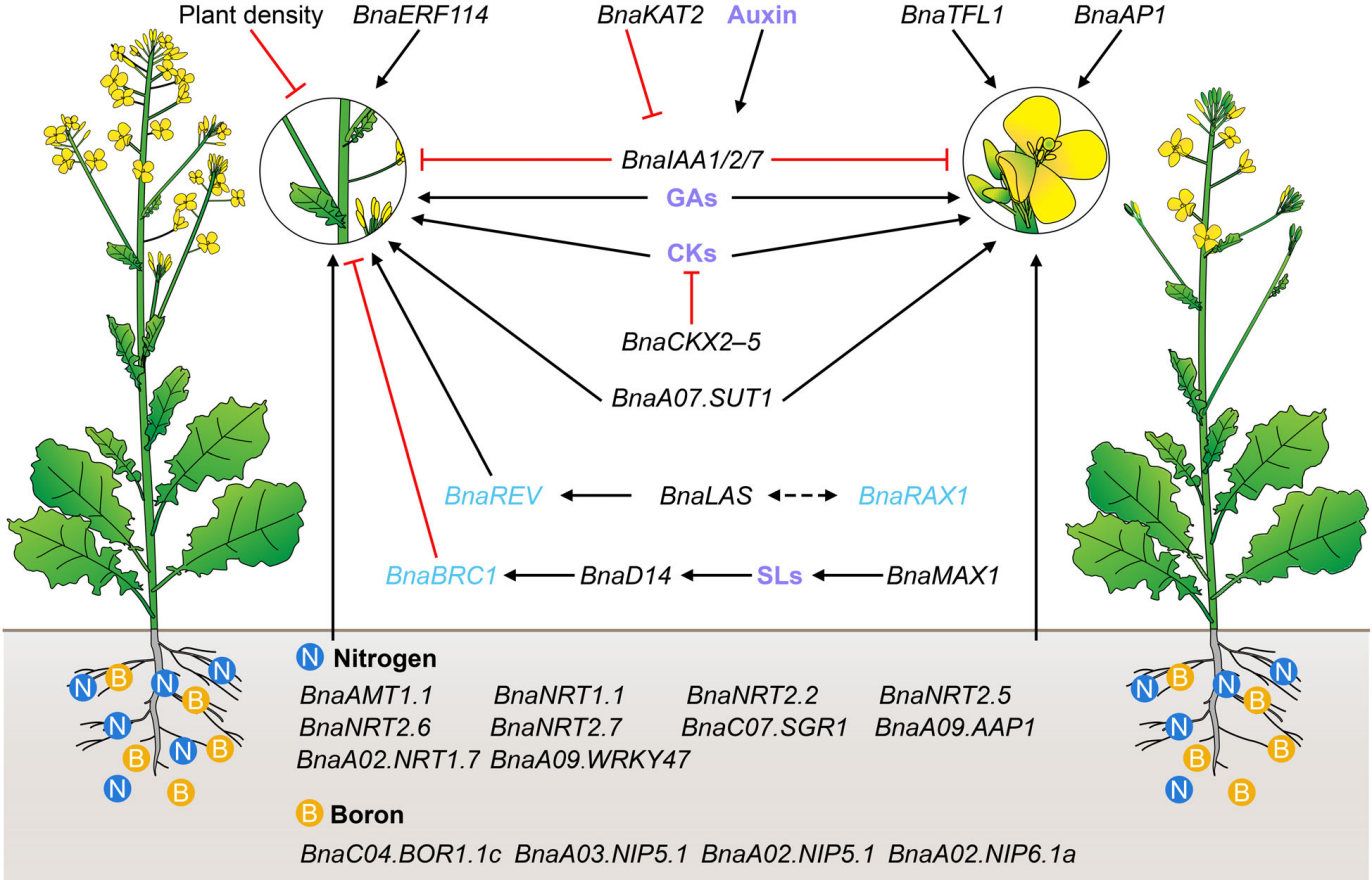
Summary of genetic, plant hormonal and environmental control of silique number in rapeseed. Black solid and dotted arrows represent direct and indirect positive regulation, respectively. Red inhibition symbols indicate negative regulation. Black and blue gene names represent genes confirmed and predicted to be involved in regulating silique number, respectively. Phytohormones are indicated in purple.

Several genes regulating silique number in rapeseed have been identified and characterized through homologous cloning (Figure 3). *BnaA02.AP1*, encoding a MADS transcription factor, is believed to be a functional homologue of *Arabidopsis AP1*, encoding a crucial regulator of FM formation (Blümel *et al*., 2015). Evidence suggests that *BnaA01.AP1* is involved in the same biological process as *AP1* and may function in a similar manner (Shah *et al*., 2018). The mutation of an *AP1* paralog in rapeseed caused substantial changes in floral morphology as well as traits related to plant architecture, such as branch number (Shah *et al*., 2018). *BnaTFL1* is a member of the phosphatidylethanolamine‐binding protein (PEBP) family, which plays an important role in determining FM identity and regulating flowering time in *Arabidopsis* and soybean (Bradley *et al*., 1997; Li *et al*., 2014). There are five copies of *BnaTFL1* in rapeseed. CRISPR/Cas9 knockout of different copies of *BnaTFL1* resulted in a substantial reduction in the number of siliques on the main inflorescence in all mutants. In addition, the *bnaa10.tfl1*, *bnaa02.tfl1*, *bnac03.tfl1*, *bnac03.tfl1*/*bnac09.tfl1* mutants show a considerable reduction in the number of branches (Sriboon *et al*., 2020). *BnaA01.ERF114* is expressed in leaf primordia, the SAM, the leaf margin meristem and reproductive organs; ectopic expression of *BnaA01.ERF114* in *Arabidopsis* resulted in shorter plant height and more branches and siliques per plant (Lyu *et al*., 2022).

*Arabidopsis LAS* is a key gene in the regulation of branch number and silique number. Ectopic expression of *BnaLAS* in *Arabidopsis* resulted in fewer branches and siliques on the main inflorescences (Yang *et al*., 2011). Branch number is an important factor affecting the number of siliques in rapeseed. Fine‐mapping based on linkage analysis identified some candidate genes that may be involved in regulating branch number in rapeseed, such as *BnaC03.BOI*, *BnaA09.ELP6*, *BnaA05.URED*, *BnaA05.ATL9* and *BnaA05.ATL38* (He *et al*., 2017; Li *et al*., 2020a; Lu *et al*., 2022; Zhang *et al*., 2018). Genome‐wide association study (GWAS) has also identified candidate genes that may regulate branch number, such as *BnaMYB83*, *BnaSPL5*, *BnaROP3*, *BnaLOF2* and *BnaCUC3* (Lu *et al*., 2017; Zheng *et al*., 2017). The functions and molecular mechanisms of these candidate genes identified by forward genetics require further verification.

## Phytohormonal control of silique number

Multiple signals, including auxin and cytokinin, play important roles in regulating the formation of both branches and floral buds (Figure 3). Auxin is biosynthesized in the apices and transported in a polar manner towards the base of the plant to repress the growth of axillary buds, whereas cytokinins promote this process (Ongaro and Leyser, 2008; Shimizu‐Sato *et al*., 2009; Wang *et al*., 2006). The location and timing of axillary bud initiation depend on auxin concentrations for the polar transport of PIN proteins (Benková *et al*., 2003; Heisler *et al*., 2005; Reinhardt *et al*., 2000, 2003; Vernoux *et al*., 2010). BnaA03.IAA7 encodes an Aux/IAA protein that is involved in improving plant architecture; its mutation increased the number of siliques per plant and improved yield in rapeseed (Li *et al*., 2019). BnaA01.KAT2, a potassium channel protein, down‐regulates the expression of *ARR3/4/6/7/9* (encoding negative regulators of cytokinin signalling) and *IAA1/2* (key genes in the auxin signalling pathway) (Yuan *et al*., 2022). Overexpressing *BnaA01.KAT2* increased the length of the main inflorescence and silique number in rapeseed.

Cytokinin oxidase/dehydrogenases (CKXs) play key roles in the irreversible degradation of cytokinin, thereby regulating plant growth and development (Figure 3). Twenty‐three *BnaCKX* genes have been identified genome‐wide in rapeseed. Transcriptome analysis showed that most *BnaCKX* genes are highly expressed in axillary buds, flowers or siliques at the reproductive growth stage, indicating that these genes are involved in regulating axillary bud, flower, and silique growth and development (Liu *et al*., 2018). Moreover, four *BnaCKX3* genes and two *BnaCKX5* genes are highly expressed in reproductive organs, and sextuple *bnackx3bnackx5* mutants have increased cytokinin concentrations in reproductive tissues, resulting in a larger and more active IM, as well increased silique number, compared with the wild type (Schwarz *et al*., 2020). Overexpressing *BnaA09.CKX2* increased thousand seed weight while decreasing silique number per plant in rapeseed (Yan *et al*., 2023). These findings suggest that cytokinin status can be modulated via mutagenesis of specific *CKX* genes to improve the yield of dicot crops such as rapeseed.

A third class of plant hormones affecting branch number is SLs, which play a negative role in bud outgrowth; this function is highly conserved in both monocots and dicots (Gomez‐Roldan *et al*., 2008; Umehara *et al*., 2008) (Figure 3). *MAX1* encodes a cytochrome P450 monooxygenase (CYP711A1) involved in SL biosynthesis (Lazar and Goodman, 2006). *Arabidopsis max1* mutants display reduced stature, increased branching and rounder rosette leaves. By contrast, overexpressing *MAX1* repressed bud outgrowth at the stem base in *Arabidopsis* (Abe *et al*., 2014; Booker *et al*., 2005; Lazar and Goodman, 2006). Rapeseed *BnaMAX1* genes have redundant functions resembling those of *Arabidopsis MAX1*, regulating plant height and axillary bud outgrowth. Simultaneous knockout of all four *BnaMAX1* alleles resulted in a semidwarf phenotype with increased branching and more siliques, contributing to improved yield per plant (Zheng *et al*., 2020). SL is perceived by *Arabidopsis* AtD14 and rice DWARF14 (OsD14), leading to the degradation of SL response regulators mediated by the F‐box protein MAX2 in *Arabidopsis* and its homologue D3 in rice (Chevalier *et al*., 2014; Jiang *et al*., 2013; Zhou *et al*., 2013). The *d14* mutant exhibits increased shoot branching with reduced plant height, similar to the SL‐deficient mutants *d10* and *max4* in *Arabidopsis* and rice, respectively (Arite *et al*., 2007; Bainbridge *et al*., 2005). Knockout of *BnaD14* in rapeseed also resulted in a semidwarf phenotype with increased branch number, contributing to increased yield (Stanic *et al*., 2021).

Brassinosteroids (BRs) are also involved in regulating branch number and FM formation (Fang *et al*., 2020; Li and He, 2020; Xu *et al*., 2020). *AtDWF4* encodes a 22α hydroxylase that functions in a rate‐limiting step in the BR biosynthetic pathway. Ectopic and organ‐specific expression of *AtDWF4* led to greater inflorescence height, branch number, number of seeds per plant and seed weight in *Arabidopsis*, tobacco (*Nicotiana tabacum*) and rice (Choe *et al*., 2001; Liu *et al*., 2007; Wu *et al*., 2008). These observations suggest that the role of *AtDWF4* is conserved in different plants (Vriet *et al*., 2013). Ectopic expression of *AtDWF4* also resulted in more branches and siliques on the main inflorescences in rapeseed (Sahni *et al*., 2016). BRs and auxin are essential regulators of plant architecture. The rapeseed *ed1* mutant shows reduced plant height, branch number and silique number per plant. *ED1* is a homologue of *AtIAA7*. BnaARF8 interacts directly with ED1 and BnaBZR1, indicating that ED1 interacts with BR signalling via BnaARF8 and BnaBZR1 to regulate plant architecture in rapeseed (Zheng *et al*., 2019).

Gibberellins (GAs) are required for the normal growth of almost all plant organs via the promotion of cell division and cell elongation. In *Arabidopsis*, GAs activate the expression of the key genes *SOC1* and *LFY*, leading to FM formation (Blazquez *et al*., 1998; Bonhomme *et al*., 2000; Moon *et al*., 2003). Endogenous GA concentrations in rapeseed are related to floral bud initiation, and external application of GA biosynthesis inhibitors inhibits flower development (Rood *et al*., 1989).

## Control of silique number by environmental stimuli

In rapeseed, silique number depends on the initiation and outgrowth of axillary buds and floral bud formation. Several environmental factors affect silique number in rapeseed, including plant population density and the availability of nitrogen, sugars and boron.

Rapeseed plants show high adaptability to changing plant density, which is an important factor affecting yield and yield components (Diepenbrock, 2000; Różyło and Pałys, 2014). SNUA (silique number per unit area) is the most important factor determining yield. Silique number per plant and branch number increase under low planting density, thus compensating for the decreased population size, while the opposite occurs at a higher planting density (Ma *et al*., 2014; Zheng *et al*., 2022). Increasing planting density can increase the competition of individual plants for nutrients and light by altering root morphology and canopy structure (Li *et al*., 2017; Ma *et al*., 2014; Rondanini *et al*., 2017). Planting density can affect photosynthesis, carbon metabolism and dry matter accumulation in rapeseed (Kuai *et al*., 2022). Planting density also affects rosette leaf diameter, which can increase the efficient capture of radiation at flowering, as well as floral branching, which can increase silique number per area (Rondanini *et al*., 2017). Therefore, increasing planting density within a certain range is a promising option for obtaining high yields in rapeseed.

Nitrogen (N) is one of the most important mineral elements for plant growth and development and a key factor in improving crop yield (Figure 3). Compared with other crops, rapeseed requires higher N fertilization for production (Rathke *et al*., 2005). N fertilizer can significantly affect rapeseed yield by altering branch number and silique number per plant (Islam and Evans, 1994). Therefore, breeding rapeseed with high N efficiency is of great strategic importance for ensuring the security of grain and oil and the sustainable development of the rapeseed industry (Zhan *et al*., 2023a). An effective way to reduce N fertilizer use is to cultivate N‐efficient cultivars identified by characterizing genotypic variation in rapeseed under contrasting N supply (Balint *et al*., 2008; Kessel *et al*., 2012; Svečnjak and Rengel, 2006). The N transporter genes *BnaAMT1.1*, *BnaNRT1.1*, *BnaNRT2.2*, *BnaNRT2.5*, *BnaNRT2.6* and *BnaNRT2.7* are expressed at markedly higher levels in the roots of N‐efficient genotypes compared to N‐inefficient genotypes, which may help explain why N‐efficient materials have more siliques and higher yields than N‐inefficient materials (Wang *et al*., 2014, 2015). In recent years, several N‐responsive genes regulating yield and yield component traits have been identified in rapeseed. The WRKY transcription factor gene *BnaA09.WRKY47* is responsive to N deficiency in rapeseed. Overexpressing *BnaA09.WRKY47* improved N reutilization in older leaves by regulating the expression of *BnaC07.SGR1*, *BnaA09.AAP1* and *BnaA02.NRT1.7*, resulting in greater silique number per plant and increased yield under low‐N conditions (Cui *et al*., 2023). Interactions between N and auxin regulate tillering and panicle branching in rice (Luo *et al*., 2020). N deficiency in rapeseed induces the expression of the auxin biosynthesis gene *BnaAUX1* and the polar auxin transporter gene *BnaPIN1*, suggesting that N might regulate silique number and branch number via auxin biosynthesis, transport and signalling pathways (Yang *et al*., 2022).

Sugars may play a role in activating bud outgrowth, while the growing stem apex may suppress branch outgrowth by acting as a sugar sink (Barbier *et al*., 2015; Kebrom, 2017). Association analysis using a diverse panel of 55 rapeseed lines identified single‐nucleotide polymorphisms in the promoter and coding sequences of the sucrose transporter gene *BnaA07.SUT1* that were significantly associated with branch number and silique number per plant (Li *et al*., 2011) (Figure 3). Elevated activities of cytosolic fructose‐1,6‐bisphosphatase (cyFBPase) and sedoheptulose‐1,7‐bisphosphatase (SBPase) are associated with higher yields in plants. Ectopic expression of the rapeseed *cyFBPase* and *SBPase* genes in tobacco resulted in greater growth and biomass and a greater number of flowers compared to wild‐type plants (Li *et al*., 2022b). Therefore, increasing the expression of *cyFBPase* and *SBPase* genes may offer an opportunity for improving yield in rapeseed.

Boron (B) is an essential micronutrient for the growth of broad bean (*Vicia faba*), barley (*Hordeum vulgare*), miscellaneous plants (Warington, 1923), rapeseed (Chen *et al*., 2018) and watermelon (*Citrullus lanatus*) (Shireen *et al*., 2020) (Figure 3). Symptoms of B deficiency include inhibited apical shoot growth, repressed root elongation, curved leaves, dried‐up floral buds and abortion of fertile pollen, which directly lead to decreases in plant productivity and reduce agricultural production and quality (Durbak *et al*., 2014; Lordkaew *et al*., 2011; Quiroga *et al*., 2020; Yang *et al*., 2013; Yuan *et al*., 2017). The uptake and translocation of B in *Arabidopsis* occur via boric acid influx channel proteins and efflux transporters (Takano *et al*., 2002, 2006, 2010). Expressing the B transporter gene *BnaC04.BOR1.1c* in the *Arabidopsis bor1‐1* mutant led to wild‐type growth and rescued the *bor1‐1* mutant phenotype. Knockdown of *BnaC04.BOR1.1c* in rapeseed increased branch number and floral organ number in the main inflorescence, but flower development was abnormal, resulting in low silique number and yield (Zhang *et al*., 2017b). *BnaA03.NIP5.1* was identified as a candidate gene for efficient B uptake in rapeseed by QTL fine‐mapping. Transgenic lines with increased *BnaA03.NIP5.1* expression exhibit improved tolerance to low B levels at both the seedling and mature stages. The *BnaA03.NIP5.1* ^*Q*^ haplotype in the natural population confers high *BnaA03.NIP5.1* expression and tolerance to low B. Field tests using a natural population and near‐isogenic lines confirmed that varieties carrying the *BnaA03.NIP5.1* ^*Q*^ allele have significantly higher silique number and yield per plant (He *et al*., 2021a). *BnaA02.NIP5.1* and *BnaA02.NIP6.1a* also positively regulate B uptake and transport (He *et al*., 2021b). Knockdown of *BnaA02.NIP5.1* or *BnaA02.NIP6.1a* in rapeseed resulted in lower B accumulation under B deficiency, leading to fewer siliques (Song *et al*., 2021). The application of B fertilizer also improves N uptake and N use efficiency, which increases branch number and silique number in rapeseed (Wang *et al*., 2022).

## Control of axillary and floral bud formation

**Figure 4:**
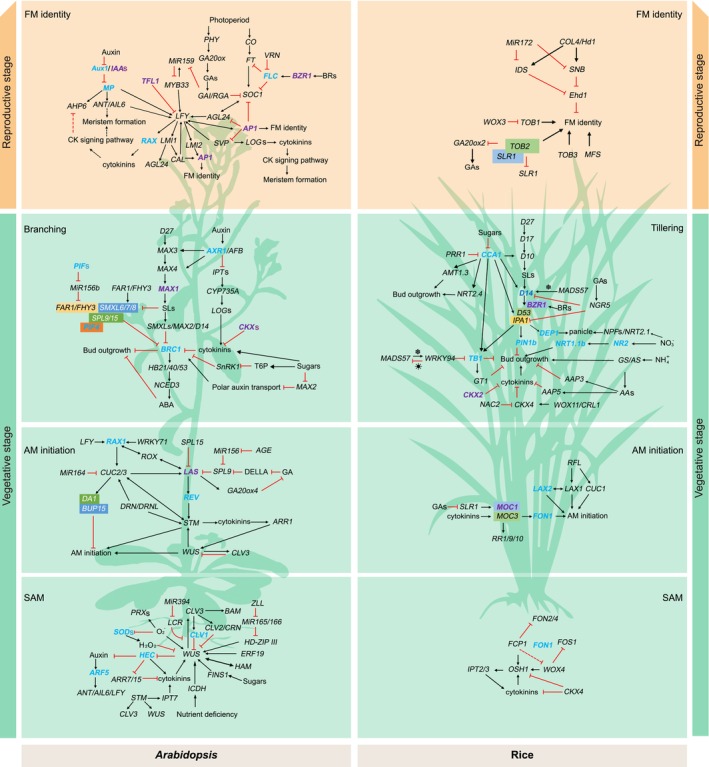
Genetic interactions of major regulatory genes that control SAM activity, shoot branching and FM formation in *Arabidopsis* and rice. From top to bottom, orange shading indicates FM formation during reproductive growth, and green shading indicates axillary bud elongation, axillary bud initiation and SAM activity during vegetative growth (*Arabidopsis* on the left and rice on the right). Adjacent solid boxes of different colours indicate gene interactions, with different colours indicating different source references describing the same step. Purple and blue gene names represent homologues of confirmed and candidate genes that regulate silique number in rapeseed. Black arrows indicate positive regulation, red lines indicate inhibition, and solid and dashed lines indicate direct and indirect regulation, respectively.

The molecular mechanisms underlying plant branching and floral bud formation have been studied extensively in many species. AM initiation and development are regulated by transcriptional regulators that are largely conserved between dicots (*Arabidopsis*/tomato) and monocots (rice), with homologous genes having the same or similar functions in different species. Therefore, the regulatory networks for branching and floral bud formation in other plants, such as *Arabidopsis*, tomato and rice, provide a reference for the regulation of these processes in rapeseed. Systematically collating information on the genetic regulatory networks of branching and floral bud formation in *Arabidopsis* and rice characterized in previous studies and reviews (Cao *et al*., 2015; Cao and Jiao, 2020; Chongloi *et al*., 2019; Fang *et al*., 2020; Han *et al*., 2014, 2020; Li *et al*., 2020c, 2023b; Luo *et al*., 2020; Mutasa‐Göttgens and Hedden, 2009; Su *et al*., 2020; Wang *et al*., 2018, 2020; Wils and Kaufmann, 2017; Zhan *et al*., 2023b; Zhang *et al*., 2022; Zhu and Wagner, 2020) allows us to shed light on the GRNs of key genes regulating silique number in rapeseed based on their homologous genes in *Arabidopsis* and rice (Figure 4; Table S4).

*BnaLAS* encodes a member of the VHIID subfamily of plant‐specific GRAS transcription factors required for AM initiation in many plants (Greb *et al*., 2003), such *Arabidopsis* (*LAS*), rice (*OsMOC1*), tomato (*Ls*) and wheat (*Triticum aestivum*; *TaMOC1*) (Greb *et al*., 2003; Li *et al*., 2003; Schumacher *et al*., 1999; Zhang *et al*., 2015). The *Arabidopsis las* mutant cannot form lateral shoots during vegetative development but forms lateral buds during the reproductive phase. During the vegetative stage, AMs initiate at a distance from the SAM and require *LAS* function; after the floral transition, AMs initiate close to the apex of the IM and do not require *LAS* (Greb *et al*., 2003). The function of *CUC2* and *CUC3* overlaps with that of *LAS*, and *LAS* expression is reduced in the *cuc2* mutant (Hibara *et al*., 2006; Raman *et al*., 2008). Cell‐type‐specific gene expression analysis and genome‐wide yeast‐one‐hybrid studies indicated that CUC2 is a positive regulator of *LAS* expression (Tian *et al*., 2014). SPL9 and SPL15, belonging to the plant‐specific SPL transcription factor family, are regulators that directly inhibit the expression of *LAS* to inhibit the initiation of AMs, thus linking the ageing pathway with AM initiation (Tian *et al*., 2014). DELLA proteins interact with SPL9 and attenuate the activity of SPL9 in repressing *LAS*, subsequently promoting the initiation of axillary buds (Yu *et al*., 2012). LAS modulates the expression of the gene encoding the GA deactivation enzyme GA2ox4 to form a low‐GA cell niche in the leaf axil region, which is required for axillary bud formation (Zhang *et al*., 2020). In addition, during vegetative development, LAS activity is required for the expression of *REVOLUTA* (*REV*; encoding a HD‐ZIPIII transcription factor) and *AUXIN RESISTANT1* (*AXR1*; encoding an E1 ligase in the RUB1 pathway) in the developing AM (Greb *et al*., 2003). *OsMOC1*, encoding a GRAS family nuclear protein, is mainly expressed in axillary buds and plays a role in axillary bud initiation and outward growth in rice (Li *et al*., 2003). *OsMOC3*, a homologue of *Arabidopsis WUS*, is involved in the initial development of the AM (Tanaka *et al*., 2015). OsMOC1 interacts with OsMOC3 and transcriptionally enhances the expression *OsFON1*, which regulates FM size and the number of all floral organs (Shao *et al*., 2019; Suzaki *et al*., 2004).

After the floral transition, the SAM is transformed into an IM. The IM either produces FMs from its flanks or remains indeterminate, iterating the pattern of inflorescence branches (Teo *et al*., 2014). *TFL1* is a key gene that affects flowering and regulates plant architecture throughout the lifecycle of plants (Conti and Bradley, 2007; Ratcliffe *et al*., 1998). The regulation of inflorescence architecture by *TFL1* is conserved among plants, including *Arabidopsis* (Ratcliffe *et al*., 1998), rice (Nakagawa *et al*., 2002), maize (*Zea mays*) (Danilevskaya *et al*., 2010), soybean (Tian *et al*., 2010) and pea (*Pisum sativum*) (Foucher *et al*., 2003). Not only does *TFL1* expression determine persistent axillary bud growth but low *TFL1* expression results in the conversion of IMs to FMs (Périlleux *et al*., 2019; Teo *et al*., 2014). Flowering and the conversion of shoot meristems to FMs occur early in *tfl1* mutants, whereas overexpression of *TFL1* led to greater inflorescence production and delayed flowering (Ratcliffe *et al*., 1998; Shannon and Meeks‐Wagner, 1991). TFL1 antagonizes the floral‐fate‐inducing factors LFY and AP1 (Denay *et al*., 2017; Wagner, 2017). FM identity genes such as *LFY* and *AP1* are ectopically expressed in the IM and bind to the *TFL1* regulatory region, and *AP1* contributes to the dissociation of the gene loop involving the 3′ distal region and transcription start site at the *TFL1* locus, which is associated with *TFL1* transcription (Kaufmann *et al*., 2010; Liu *et al*., 2013; Moyroud *et al*., 2011; Winter *et al*., 2011). Up‐regulation of *LFY* and *AP1* is delayed in plants overexpressing *TFL1* during the floral transition (Bowman *et al*., 1993; Bradley *et al*., 1997; Prusinkiewicz *et al*., 2007; Ratcliffe *et al*., 1998). TFL1 is recruited to *LFY* chromatin and regulates *LFY* expression with the help of the bZIP transcription factor FD (Goretti *et al*., 2020; Zhu *et al*., 2020). TFL1 negatively regulates FD‐dependent transcription of its target genes to fine‐tune flowering and IM development (Hanano and Goto, 2011). The expression of *TFL1* is inhibited by SOC1, SVP, AGL24 and SEP4 in the emerging FM (Liu *et al*., 2013).

OsTFL1 represses flowering and controls inflorescence architecture in rice. The molecular mechanism of the phase transition and inflorescence architecture is conserved between grass and dicot species (Nakagawa *et al*., 2002). All four *RCN* genes (rice *TFL1‐like* genes) are predominantly expressed in the vasculature, and RCN proteins are transported to the shoot apex, where they antagonize florigen activity and regulate inflorescence development. The antagonistic activity of RCN against *Hd3a*, a rice homologue of *FT*, depends on its 14‐3‐3 binding activity (Kaneko‐Suzuki *et al*., 2018). OsMADS34 promotes the transition from the vegetative stage to the reproductive stage and IM development (Kobayashi *et al*., 2012), as well as spikelet meristem formation (Kobayashi *et al*., 2010). The *osmads34* mutant exhibits altered inflorescence morphology, with an increase in the primary branch number and a decrease in the secondary branch number and seed‐setting rate (Gao *et al*., 2010; Zhang *et al*., 2016). The repression of *RCN4* by OsMADS34 primarily functions to counterbalance secondary branch meristem identity and promote the transition to spikelet meristem identity (Zhu *et al*., 2022).

*AP1* and *LFY* are key genes in the transition of an IM into an FM. *AP1* is a key target of LFY, a MADS box transcription factor that functions in the primordium to determine the fate of the IM for flowering (Bowman *et al*., 1993; Wagner *et al*., 1999). The establishment of FM identity and subsequent flower development are largely dependent on the activity of the transcription factors LFY and AP1 (Grandi *et al*., 2012; Pastore *et al*., 2011; Saddic *et al*., 2006; Wils and Kaufmann, 2017). *LFY* is expressed before the formation of the first floral primordium, and *AP1* is subsequently up‐regulated in the young floral primordium, which determines the fate of flower formation (Blázquez *et al*., 1997; Hempel *et al*., 1997; Mandel *et al*., 1992; Yamaguchi *et al*., 2009). LFY, through a series of coherent and logical feed‐forward loops, directly or indirectly up‐regulates *AP1* (Pastore *et al*., 2011; Wagner *et al*., 1999; Yamaguchi *et al*., 2014). During FM development, AP1 down‐regulates several floral repressor genes of the *AP2* family of transcription factors such as *SNZ*, *TOE1* and *TOE3* (Kaufmann *et al*., 2010) and multiple florals promoting transcription factor genes such as *AGL24*, *SOC1*, *FD*, *FUL*, *SVP* and *TFL1* to promote the continuous emergence of floral primordia (Hanano and Goto, 2011; Kaufmann *et al*., 2010; Liljegren *et al*., 1999; Wigge *et al*., 2005). In the above regulatory pathways, AP1 induces *LFY* expression through a positive feedback loop to establish the identity of FM, ensuring its expression for downstream floral organ speciation and development while maintaining its determinism (Grandi *et al*., 2012). The repression of *SVP* and *AGL24* by AP1 relieves the negative regulation of *SEP3* (Posé *et al*., 2012), leading to the formation of AP1/SEP3 heterodimers and inducing the expression of *AP3* and *PI*, thereby initiating the development of floral organs (Gregis *et al*., 2008; Wils and Kaufmann, 2017).

SLs are key phytohormones that regulate the growth of lateral meristems in plants. The role of MAX1 in regulating SL biosynthesis is functionally conserved in rice, *Arabidopsis*, wheat, maize and rapeseed (Al‐Babili and Bouwmeester, 2015; Booker *et al*., 2005; Cardoso *et al*., 2014; Sigalas *et al*., 2023; Yoneyama *et al*., 2018; Zheng *et al*., 2020). MAX1 converts carlactone (CL) to carlactonoic acid (CLA) (Abe *et al*., 2014). A SABATH methyltransferase then catalyses the conversion of CLA to Me‐CLA in *Arabidopsis* (Wakabayashi *et al*., 2021). Me‐CLA is a non‐canonical SL that interacts with AtD14, a receptor protein in the SL signalling pathway, indicating that it is biologically active in suppressing shoot branching (Abe *et al*., 2014). SL induces changes in the structure of AtD14 protein and is then hydrolysed into CLIM (covalently linked intermediate molecule), promoting an interaction between AtD14 and AtD3 (Yao *et al*., 2018). AtD14 may also play a role in SL signal transduction pathways. The mutation of *OsD14* increases tiller number and decreases plant height in rice (Arite *et al*., 2009; Liu *et al*., 2009), and the transcription of *OsD14* is repressed by transcription factors such as OsMADS57 and OsCCA1. OsMADS57 interacts with OsTB1, reducing the inhibition of *OsD14* transcription by OsMADS57 (Guo *et al*., 2013). OsCCA1 positively regulates the expression of *OsTB1*, *OsD14* and *IPA1*, thus repressing tiller‐bud outgrowth (Wang *et al*., 2020). *OsD14* is associated with *OsD3* in a GR24‐dependent manner, suppressing shoot branching in rice (Zhao *et al*., 2014).

## GRNs of key genes for silique number

**Figure 5:**
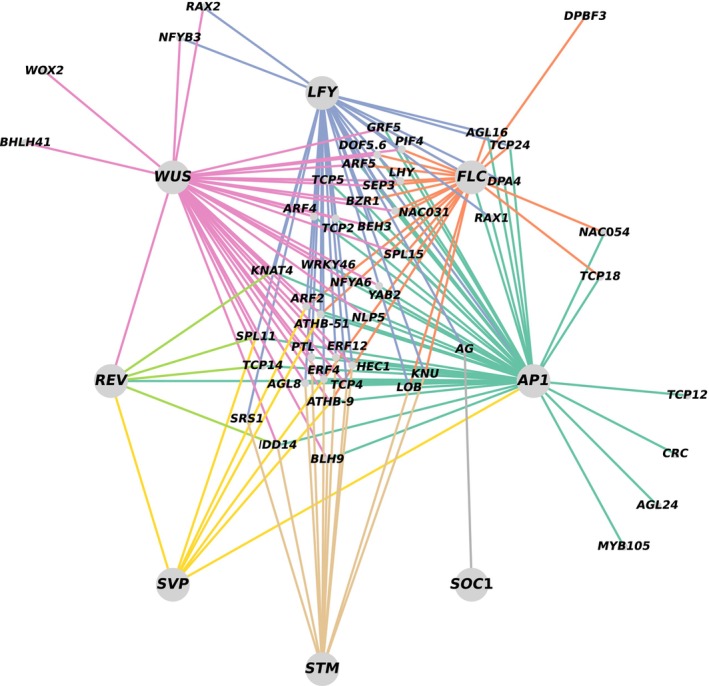
Gene regulatory network for silique number in *Arabidopsis*. The 54 genes in the genetic regulatory network were derived from the intersection of *Arabidopsis* homologues of transcription factor gene among candidate genes and eight genes (*WUS*, *LFY*, *FLC*, *AP1*, *SOC1*, *STM*, *SVP* and *REV*) using ChIP‐seq. Large and small grey dots represent genes identified by ChIP‐seq and target genes, respectively. Different coloured lines connecting different genes indicate transcriptional regulation of target genes by different transcription factors.

Transcriptional regulation is important for plant growth and development; the downstream target genes of transcription factors can be identified through ChIP‐seq (Schmidt *et al*., 2009). We therefore compiled a list of the downstream target genes of the key transcription factors WUS (Ma *et al*., 2019), AP1 (Goslin *et al*., 2017; Kaufmann *et al*., 2010; Pajoro *et al*., 2014; Winter *et al*., 2015), LFY (Goslin *et al*., 2017; Jin *et al*., 2021; Moyroud *et al*., 2011; Sayou *et al*., 2016; Winter *et al*., 2015; Zhu *et al*., 2020), STM (Li *et al*., 2018), FLC (Deng *et al*., 2011; Mateos *et al*., 2017), REV (Brandt *et al*., 2012), SVP (Gregis *et al*., 2013; Mateos *et al*., 2015, 2017; Tao *et al*., 2012) and SOC1 (Immink *et al*., 2012; Tao *et al*., 2012) identified by ChIP‐seq (Table S5). The 13 793 target genes included 146 candidate genes for silique number. We used 54 transcription factor genes identified from among the 146 candidate genes as well as the eight key transcription factors to construct a GRN (Figure 5; Table S5). This analysis revealed several regulatory pathways, such as the direct binding of LFY to the promoter of *AP1* to regulate its expression (Bowman *et al*., 1993; Wagner *et al*., 1999). However, a large number of downstream genes that may regulate axillary bud initiation and elongation and control floral bud formation remain unclear. The GRN reveals some important genes that control silique number. For example, *TEM1* (AT1G25560) was enriched in the ChIP‐seq data for the eight key transcription factors. The potential regulatory relationships revealed by *Arabidopsis* ChIP‐seq data provide a reference for studying the genetic regulatory mechanism of silique number in rapeseed.

## Summary and future perspectives

### Identification of genes and loci for the genetic improvement of silique number

Silique number is a complex quantitative trait in rapeseed. Hundreds of QTLs regulating silique number have been identified; however, few genes responsible for controlling silique number have been cloned. This may be due to the low heritability of rapeseed silique number and the lack of omics evidence. It is still challenging to identify the key genes regulating silique number in rapeseed by forward genetics. The release of several reference genomes, such as Darmor v4.1 and v10 (Chalhoub *et al*., 2014; Rousseau‐Gueutin *et al*., 2020) and ZS11.v0, v10 and v2 (Chen *et al*., 2021; Song *et al*., 2020; Sun *et al*., 2017), and the application of SNP chips (Edwards *et al*., 2013; Li *et al*., 2023a; Xiao *et al*., 2021) in rapeseed provide opportunities for discovering key genes controlling silique number in rapeseed. In addition, several QTL mapping methods have been developed for identifying candidate genes associated with important agronomic traits in rapeseed, such as natural‐population‐based GWAS (He *et al*., 2017; Khan *et al*., 2021), transcriptome‐wide association analysis (TWAS) (Tan *et al*., 2022; Tang *et al*., 2021) and parental‐population‐based linkage analysis methods such as bulked segregant RNA sequencing (BSR) (Fu *et al*., 2019) and bulked segregant analysis (BSA) (Wang *et al*., 2016; Yu *et al*., 2023; Zhao *et al*., 2020). Databases built upon these data, such as A Gene Expression Database for Brassica Crops (BrassicaEDB, https://brassica.biodb.org/) (Chao *et al*., 2020), qPCR Primer Database (qPrimerDB, https://qprimerdb.biodb.org/) (Lu *et al*., 2018), Brassicaceae Database (BRAD, http://www.brassicadb.cn/#/) (Chen *et al*., 2022) and *Brassica napus* multi‐omics information resource (BnIR, https://yanglab.hzau.edu.cn/BnIR) (Yang *et al*., 2023), provide important data resources and analysis platforms for genetic breeding research in rapeseed. Advances in QTL mapping methods and multi‐omics data analysis will help overcome the difficulties in cloning key genes regulating silique number in rapeseed.

### Dissection of the molecular mechanism underlying silique number to enable its genetic improvement

Silique number in rapeseed is mainly determined by the initiation and elongation of axillary buds and the formation of floral buds. Axillary bud initiation and elongation and floral bud formation are very complex biological processes, but some progress has been made in understanding their mechanisms in *Arabidopsis* and rice. Homologous cloning‐based approaches in rapeseed have identified several genes that regulate silique number, such as *BnaMAX1*, *BnaLAS*, *BnaTFL1* and *BnaAP1*. However, these genes are not sufficient for the genetic improvement of silique number. As rapeseed is an allotetraploid, genetic regulation is more complex in rapeseed than in *Arabidopsis* and rice, with not only simple functional redundancy but also possible subfunctionalization between homologous genes (Babula‐Skowrońska *et al*., 2015; Schiessl, 2020; Schiessl *et al*., 2019). RNA interference (RNAi) and CRISPR‐based editing are important tools for studying gene function. RNAi provides an opportunity to develop novel traits in transgenic plants and shows high specificity, high efficiency and reliability for studying gene function (Fishilevich *et al*., 2016; Ramon *et al*., 2014). CRISPR‐based editing can target a single gene as well as multiple orthologous genes, such as the five *BnaTFL1* gene copies; CRISPR/Cas9 gene editing technology has been used to generate different mutants such as *bnac03.tfl1a*, *bnaa02.tfl1* and *bnac03.tfl1/bnac09.tfl1* (Sriboon *et al*., 2020). More importantly, mutants generated by CRISPR editing can be used directly in crop production or as pre‐breeding materials (Hussain *et al*., 2018; Zhang *et al*., 2017a). Therefore, CRISPR‐based editing is a powerful tool for analysing key genes related to silique number in rapeseed.

### Potential application of silique number molecular mechanism in rapeseed breeding

Genetic improvement of silique number is very important in improving rapeseed yield. In this study, twenty‐two regions were identified, some of which were associated with one trait only, such as *qSNRT_A03.3* for silique number, and some regions associated with multiple traits, such as *qSNRT_A03.2* for silique number and branch number. These regions that regulate multiple traits may have pleiotropic candidate genes or linkage between two genes that regulate silique number and branch number, respectively. The development of molecular markers for twenty‐two regions could be helpful in genetic improvement of silique number‐related traits.

Haplotype‐assisted breeding is an important approach for high‐yield breeding in rapeseed. At present, a number of silique number candidate genes have been identified in rapeseed, including 240 silique number candidate genes predicted in this study, and some branch number candidate genes were excavated by linkage analysis and association analysis (He *et al*., 2017; Li *et al*., 2020a; Lu *et al*., 2017, 2022; Zhang *et al*., 2018; Zheng *et al*., 2017). The excellent haplotype of these candidate genes will be helpful to silique number improvement and increase yield in rapeseed.

At present, although, our knowledge of the molecular mechanisms regulating traits related to silique number is limited. It is known that gene regulation of silique number could provide some ways for yield improvement. The *BnaD14* is strigolactone signalling pathway genes in rapeseed. Mutation *bnad14* could notably increase the number of branches in rapeseed, but it has no remarkable effect on increasing yield (Stanic *et al*., 2021). *BnaCKX3* and *BnaCKX5* are a group of enzymes that regulate oxidative cleavage to maintain cytokinin homeostasis. Sextuple *bnackx3bnackx5* mutants have increased cytokinin concentrations in reproductive tissues, resulting in a larger and more active IM, as well increased silique number (Schwarz *et al*., 2020). Therefore, simultaneous knockout of *BnaD14*, *BnaCKX3* and *BnaCKX5* in rapeseed may increase branch number, increase silique number per branch and greatly improve rapeseed yield. However, breeding rapeseed with ideal architecture requires the dissection of genetic loci associated with traits related to silique number or the cloning of the underlying genes (Liu *et al*., 2022). The application of multi‐omics including genome, transcriptome, and epigenome and advanced technologies such as GWAS and CRISPR‐based editing in rapeseed will facilitate the identification of key genes controlling silique number. As more regulatory genes are cloned and studied, the genetic networks controlling silique number in rapeseed will be revealed, paving the way for improving rapeseed yield by increasing silique number.

## Author contributions

KL: Conceptualized the study and performed the methodology. HW: Planned, designed and wrote the original draft. XL: Drew the figures and designed and revised the manuscript. BM: Drew the figures. SU Khan, MQ and YF: Revised the manuscript. MZ and HY: Collected the data for QTLs and QTNs. All authors read and approved the final manuscript.

## conflict of interest

The authors declare that they have no known competing financial interests or personal relationships that could have appeared to influence the work reported in this review.

## Supporting information


**Table S1** List of QTLs/QTNs for silique number in rapeseed identified in previous studies.

**Table S2** Candidate genes for silique number in rapeseed detected in overlapping QTL regions.

**Table S3** List of rapeseed homologues of *Arabidopsis* and rice genes involved in controlling silique number or grain number.

**Table S4** *Arabidopsis* and rice orthologs of candidate genes in rapeseed.

**Table S5** List of potential downstream target genes of the key transcription factors WUS, STM, SOC1, FLC, REV, SVP, LFY, and AP1 in *Arabidopsis* identified by ChIP‐seq in previous studies.

## Data Availability

Data sharing is not applicable to this article as no new data were created or analyzed in this study.
